# Novel dual-layer internal limiting membrane flap with superior-inferior coverage combined with autologous blood application for large macular hole: anatomical and functional outcomes

**DOI:** 10.3389/fmed.2026.1693800

**Published:** 2026-02-19

**Authors:** Li-Hua Zhang, Min Lin, Pei-Quan Zhao, Ping Fei

**Affiliations:** 1 Department of Ophthalmology, Binzhou Hubin Aier Eye Hospital, Binzhou, China; 2 Department of Ophthalmology, Xinhua Hospital, Affiliated to Shanghai Jiao Tong University School of Medicine, Shanghai, China

**Keywords:** internal limiting membrane flap, macular hole, post-surgery effect, surgery, treatment

## Abstract

**Purpose:**

To evaluate the efficacy of a novel dual-layer internal limiting membrane (ILM) flap with superior-inferior (S-I) coverage combined with autologous blood tamponade for the treatment of large macular hole (MH) > 400 μm in diameter.

**Methods:**

This retrospective interventional case series included 21 eyes from 21 patients who underwent vitrectomy with the dual-layer ILM flap with S-I coverage combined with autologous blood tamponade between February 2022 and July 2024. The surgical procedure involved creating superior and inferior ILM flaps that were inverted to achieve dual-layer coverage over the MH, followed by application of autologous whole blood and 15% C3F8 gas tamponade. Primary outcomes included anatomical closure rate assessed by optical coherence tomography (OCT) and best-corrected visual acuity (BCVA) improvement at 3-month follow-up.

**Results:**

All 21 eyes (100%) achieved complete MH closure, including cases with a mean baseline diameter of 717.29 ± 127.00 μm (range: 521–949 μm). Median logMAR BCVA significantly improved from 1.70 (IQR, 1.30–1.70) to 0.82 (IQR, 0.61–0.82) after the surgery (*p* < 0.001). No sight-threatening complications were observed during the follow-up period.

**Conclusion:**

The dual-layer ILM flap with S-I coverage showed excellent anatomical and functional outcomes in large MH, indicating that this technique may be an effective surgical approach for challenging cases. Further randomized controlled trials are needed to validate these preliminary findings.

## Introduction

Macular hole (MH) is a vision-threatening condition characterized by a full-thickness defect in the foveal retina, leading to central visual impairment (1, 2). Since Kelly and Wendel first introduced vitrectomy with internal limiting membrane (ILM) peeling as a standard surgical treatment for MH in 1991, this technique has significantly improved closure rates and visual outcomes (3). The rationale behind ILM removal is to eliminate tangential traction forces that contribute to MH formation and persistence (4). Despite high success rates with conventional ILM peeling (5, 6), challenges remain in achieving anatomical closure, particularly for large (>400 μm) or chronic MH. These cases often exhibit lower closure rates and poorer visual recovery due to insufficient glial cell proliferation and inadequate tissue bridging across the hole (7). To address this limitation, modified surgical techniques have been developed, including the inverted ILM flap approach introduced by Michalewska, which demonstrated improved closure rates for large MH by providing a scaffold for glial cell migration (8). Building upon this concept, we have developed a novel dual-layer ILM flap with superior-inferior (S-I) coverage. In this procedure, the peeled ILM is inverted and meticulously positioned over the MH in a dual-layer configuration to enhance the structural support. To further stabilize the flap, autologous whole blood is applied during surgery; upon clot formation, it serves as a natural bio-adhesive that helps secure the ILM flap in place and supports early tissue bridging (9, 10).

The primary objective of this study is to evaluate the efficacy of the dual-layer ILM flap technique, thereby providing novel evidence-based insights for optimizing ILM manipulation strategies in macular hole surgery.

## Materials and methods

We retrospectively collected and observed the medical data of 21 eyes from 21 patients with large MH, who have undergone the dual-layer ILM flap with S-I coverage combined with autologous blood application surgery in our institution between February 2022 and July 2024. All surgeries were performed by an experienced surgeon.

Patients with full-thickness macular hole measuring > 400 μm in minimum diameter were included. The exclusion criteria comprised: (1) patients with any prior ocular surgery history except uncomplicated cataract surgery; (2) those presenting with any other ocular comorbidity that could potentially confound the assessment of postoperative visual outcomes, including but not limited to advanced glaucoma, optic neuropathies, amblyopia, significant corneal opacity, or concomitant vitreoretinal pathologies. This study was conducted in accordance with the Declaration of Helsinki. Written informed consent was obtained from all participants for participation in the study and for the publication of any potentially identifiable images or data included in this article.

### Ophthalmic examination and measurement

All patients underwent a comprehensive ophthalmic examination preoperatively and at the final postoperative follow-up (minimum 3 months). The BCVA was measured using a standard Early Treatment Diabetic Retinopathy Study (ETDRS) chart at a distance of 4 meters and converted to Snellen notation. For statistical analysis, Snellen BCVA was converted to the logarithm of the minimum angle of resolution (logMAR) values.

Optical coherence tomography (OCT) imaging was performed using the Spectralis OCT PLUS (Heidelberg, Germany). OCT was performed with high-density horizontal and radial scans centered on the fovea. The minimum linear diameter (MLD) of the macular hole was measured on B-scan images as the narrowest distance between the edges of the neurosensory retina at the level of the hole (11, 12), using the device’s caliper tool. MLD measurements by OCT have been shown to be reliable and clinically relevant in evaluating macular hole size and surgical planning (13).

### Surgical procedures

#### Pars plana vitrectomy

Standard 23-gauge, three-port pars plana vitrectomy was performed under retrobulbar anesthesia. For phakic patients with concurrent cataracts, phacoemulsification with intraocular lens implantation was conducted prior to vitrectomy surgery.

#### Dual-layer ILM flap with S-I coverage

**FIGURE 1:**
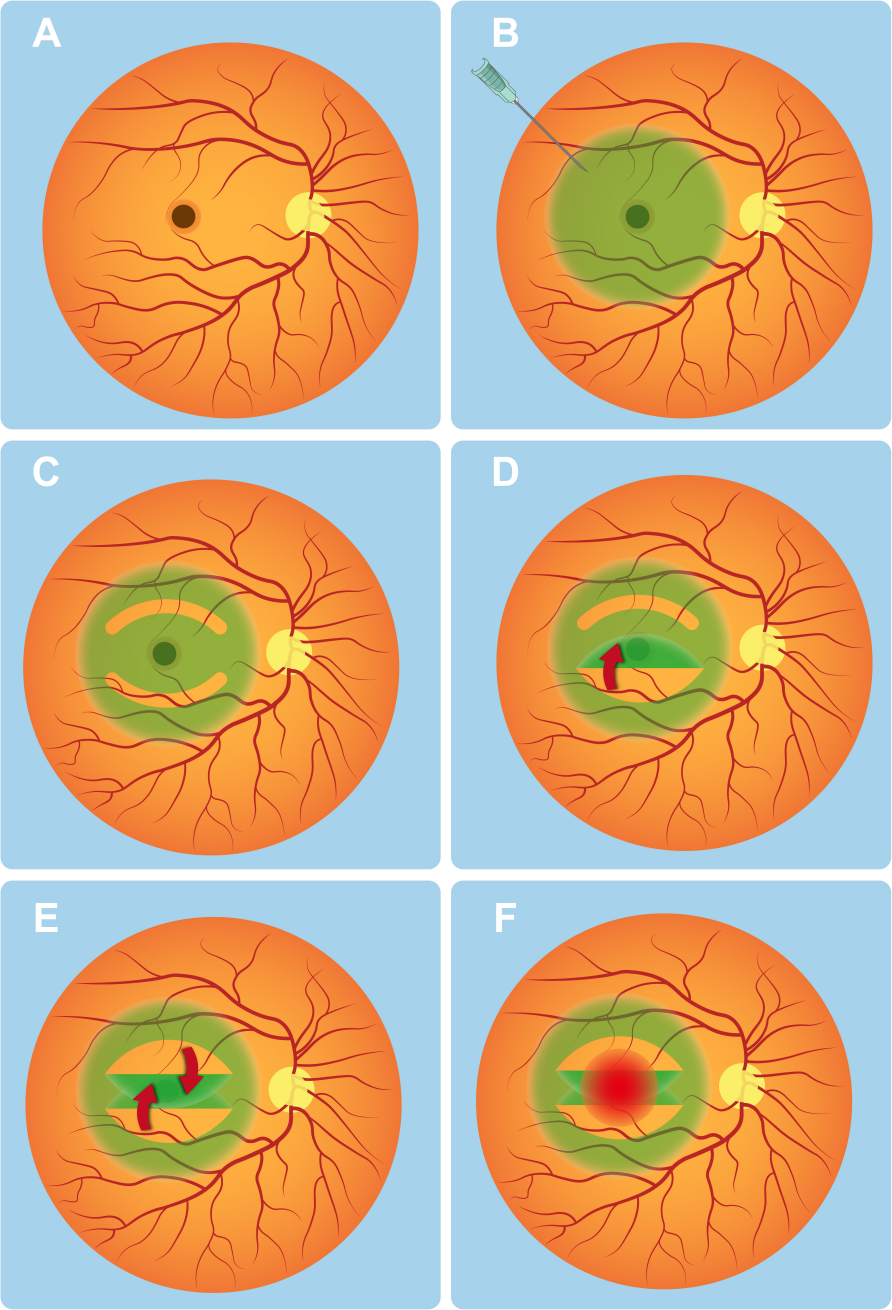
Schematic demonstration of dual-layer ILM flap with S-I coverage for macular hole repair. **(A)** Schematic representation of a full-thickness macular hole. **(B)** Intraoperative view after indocyanine green (0.125%) staining of the ILM. **(C)** Superior/inferior arcuate disinsertions of ILM created by blunt dissection from the fovea. **(D)** Inferior ILM flap inverted centripetally over the macular hole (red arrow indicates flap trajectory). **(E)** Superior ILM flap subsequently overlapped in crisscross fashion (red arrow shows secondary positioning). **(F)** Final appearance after application of autologous whole blood over the inverted flaps.

Figure 1A presents a schematic representation of macular hole. Following vitrectomy, the ILM was then stained with 0.125% indocyanine green (ICG) solution under fluid (Figure 1B). Using 25-gauge forceps, the macular epiretinal membrane was carefully peeled off. The ILM was then circumferentially incised, with the superior flap extending approximately 3 MH diameters from its base and the inferior flap measuring 2 MH diameters in length (Figure 1C), ensuring the superior flap was larger than the inferior flap to facilitate complete coverage.

Subsequently, the inferior ILM flap was inverted first, followed by the superior flap, which was carefully positioned to fully overlap the inferior flap, achieving dual-layer closure of the MH (Figures 1D,E).

#### Application of autologous whole blood

Following the dual-layer ILM Flap procedure, any excess portion of the superior flap beyond the inferior flap was secured using autologous whole blood to enhance adhesion and stability (Figure 1F). Peripheral venous blood was aseptically collected and gently instilled over the macular hole to cover the inverted ILM flaps. After 15 s of undisturbed contact, the formed blood clot effectively sealed the macular defect.

#### Intraocular tamponade

At the conclusion of the surgical procedure, 15% perfluoropropane (C3F8) gas was administered for intraocular tamponade.

#### Postoperative management

Patients were instructed to maintain a strict face-down positioning for 2 weeks postoperatively.

### Closure assessment and classification

Postoperative macular hole closure was assessed using OCT. Anatomical closure was defined as the absence of bare retinal pigment epithelium (RPE) exposure to the vitreous chamber and the presence of tissue continuity over the fovea, irrespective of the foveal contour shape or the type of bridging tissue. This definition follows the updated classification proposed by Rossi et al. (14), which categorizes closure patterns based on retinal layer restoration and the presence of filling tissue. According to this system, successful closures are classified as Type 1 (reconstitution of retinal layered anatomy) or Type 2 (closure with autologous or heterologous tissue interrupting the normal foveal architecture), while Type 0 denotes an open macular hole.

### Statistical analysis

For statistical analysis, the BCVA measured in Snellen notation was converted to the logarithm of the minimum angle of resolution (logMAR) (15, 16). Counting fingers (CF) visual acuity was standardized to logMAR equivalents of 2.0 (17, 18). The Shapiro-Wilk test was used to assess the normality of the data distribution. Since the data were found to be non-normally distributed (*p* < 0.001), preoperative and postoperative BCVA were compared using the nonparametric Wilcoxon signed-rank test. All statistical analyses were performed using SPSS (Version 27.0, United States), with a *p* < 0.05 considered statistically significant.

## Results

### Demographic and clinical characteristics

A total of 21 eyes from 21 consecutive patients were enrolled in this retrospective interventional case series. The baseline demographic and clinical characteristics of the patients, along with the surgical outcomes, are summarized in Tables 1, 2, respectively. The mean age was 68.2 ± 5.6 years (range: 59–80), with a female predominance (85.7%). The mean preoperative MLD of the macular holes, as measured by OCT, was 717.29 ± 127.00 μm (range: 521–949 μm). Concomitant cataract was present in 20 eyes (95.2%).

**TABLE 1:** Baseline clinical factors of patients.

Variables	*N* = 21
Age, years	68.19 ± 5.62 (rang: 59–80)
**Gender**	
Male	3(14.29%)
Female	18(85.71%)
**Eye**	
Right	11(52.38%)
Left	10(47.62%)
**Lens status**	
Cataract	20(95.24%)
IOL	1(4.76%)

N, number; IOL, intraocular lens.

**TABLE 2:** Patient characteristics and surgical outcomes.

No	Age	Sex	Predisposing disease	Size of MH before the surgery, μ m	MH status after the surgery	Preoperative BCVA-Snellen (logMAR)	Postoperative BCVA-Snellen (logMAR)	Follow-up duration (months)
1	67	F	Cataract with large MH	812	Closed	20/667(1.5)	20/67(0.5)	6
2	71	F	Cataract with large MH	658	Closed	20/400(1.3)	20/100(0.7)	6
3	59	F	Cataract with large MH	614	Closed	20/1,000(1.7)	20/50(0.4)	9
4	67	F	Cataract with large MH	760	Closed	20/2,000(2)	20/133(0.8)	6
5	74	F	Cataract with large MH	521	Closed	20/2,000(2)	20/133(0.8)	6
6	64	F	Cataract with large MH	862	Closed	20/2,000(2)	20/400(1.3)	9
7	70	F	Cataract with large MH	830	Closed	20/2,000(2)	20/133(0.8)	6
8	65	M	Cataract with large MH	671	Closed	20/400(1.3)	20/100(0.7)	6
9	66	F	Cataract with large MH	579	Closed	20/1,000(1.7)	20/67(0.5)	6
10	61	F	Cataract with large MH	719	Closed	20/400(1.3)	20/50(0.4)	6
11	74	F	Cataract with large MH	625	Closed	20/1,000(1.7)	20/133(0.8)	7
12	66	F	Cataract with large MH	915	Closed	20/200(1.0)	20/100(0.7)	6
13	73	M	Cataract with large MH	886	Closed	20/1,000(1.7)	20/133(0.8)	6
14	69	M	Cataract with large MH	785	Closed	20/400(1.3)	20/133(0.8)	8
15	61	F	Cataract with large MH	535	Closed	20/400(1.3)	20/133(0.8)	6
16	72	F	IOL with large MH	839	Closed	20/1,000(1.7)	20/133(0.8)	7
17	69	F	Cataract with large MH	801	Closed	20/1,000(1.7)	20/100(0.7)	6
18	80	F	Cataract with large MH	672	Closed	20/400(1.3)	20/100(0.7)	6
19	69	F	Cataract with large MH	634	Closed	20/400(1.3)	20/133(0.8)	6
20	59	F	Cataract with large MH	949	Closed	20/1,000(1.7)	20/200(1.0)	7
21	76	F	Cataract with large MH	685	Closed	20/400(1.3)	20/33(0.2)	24

F, female; M, male; MH, macular hole; IOL, intraocular lens; BCVA, best-corrected visual acuity; logMAR, logarithm of the minimum angle of resolution.

### Surgical procedures and follow-up

All patients underwent pars plana vitrectomy combined with the novel dual-layer ILM flap technique with S-I coverage, followed by autologous whole blood application, as detailed in the Methods section. Concurrent phacoemulsification with intraocular lens (IOL) implantation was performed in 20 eyes. The mean postoperative follow-up duration was 7.38 ± 3.93 months, which allowed for robust assessment of both anatomical and functional outcomes.

### Anatomical outcomes

**FIGURE 2:**
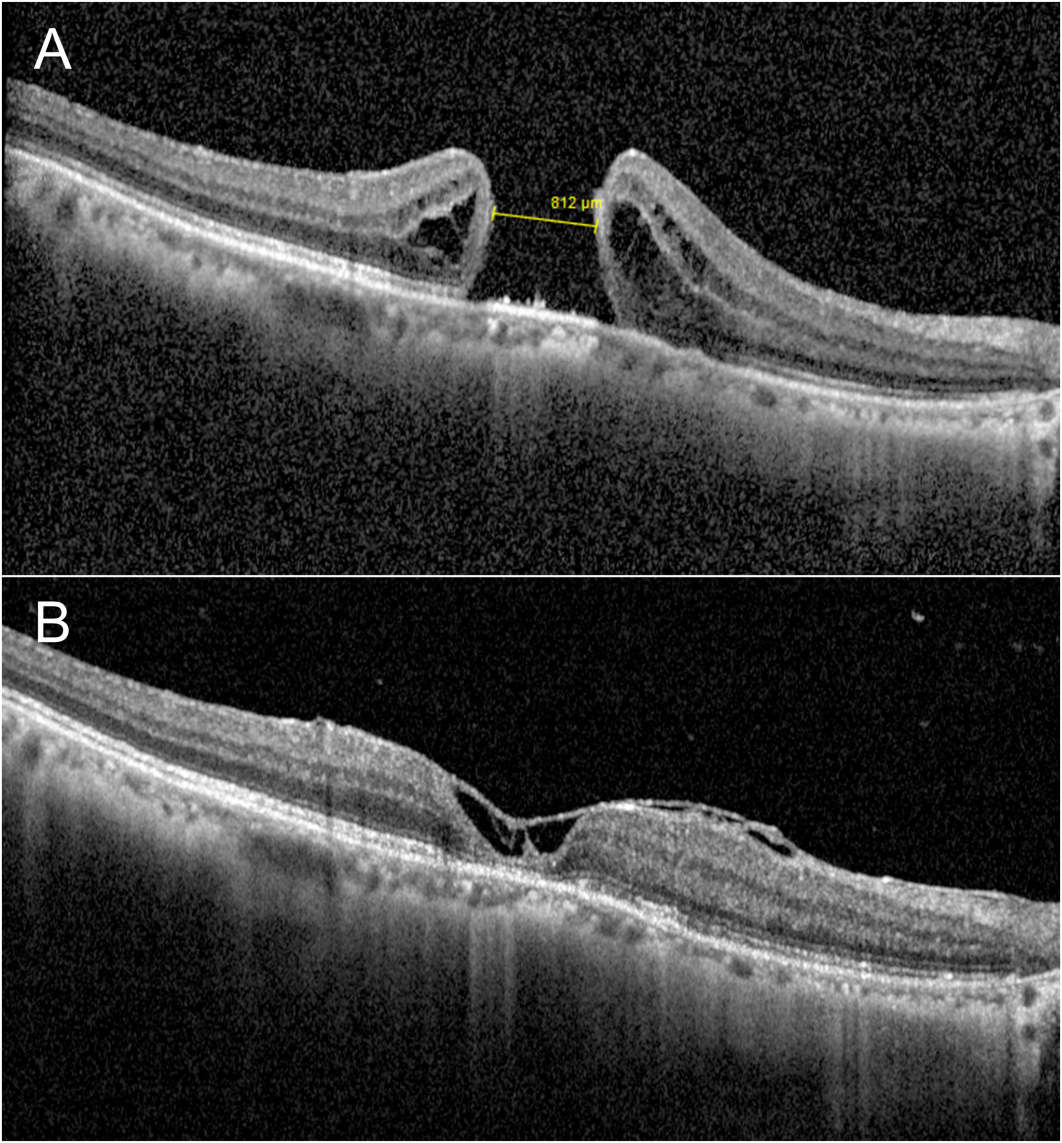
Case 1: A 67-year-old female patient presented with a 2-month history of visual decline. Preoperative examination confirmed a full-thickness macular hole (diameter: 812 μm, as measured by OCT; **A**) with concurrent cataracts. The patient underwent combined surgery (phacoemulsification with intraocular lens implantation + pars plana vitrectomy + dual-layer ILM flap with S-I coverage + autologous blood tamponade). Postoperative OCT at 1 month demonstrated complete hole closure **(B)**. At the final follow-up visit (6 months postoperatively), the BCVA improved significantly from 20/667 preoperatively to 20/67.

All 21 eyes (100%) achieved complete anatomical closure of the macular hole at the final follow-up (representative case shown in Figure 2). According to the updated classification system by Rossi et al. (14), all successfully closed holes in this series were classified as Type 1, indicative of reconstitution of the retinal layered anatomy. No cases demonstrated features of Type 2 closure or Type 0.

### Functional outcomes

Postoperative best-corrected visual acuity (BCVA) improved significantly in all eyes. The median preoperative BCVA was 1.70 logMAR (interquartile range (IQR), 1.30–1.70), which improved to 0.82 logMAR (IQR, 0.61–0.82) at the final follow-up. This improvement was statistically significant (Wilcoxon signed-rank test: *Z* = –4.022, *p* < 0.001), with a large effect size (*r* = 0.88), indicating a marked enhancement in visual function.

### Correlation analysis

**FIGURE 3:**
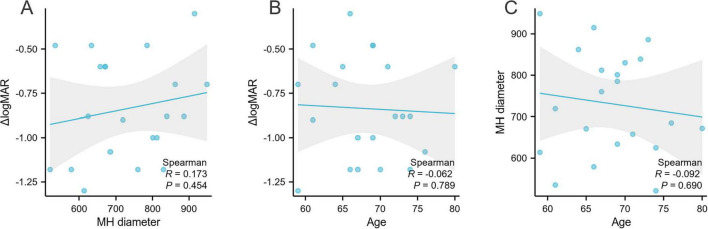
Spearman correlation analyses examining the relationships between macular hole (MH) diameter, patient age, and visual improvement measured by the change in logarithm of the minimum angle of resolution (ΔlogMAR). **(A)** Correlation between MH diameter and ΔlogMAR. **(B)** Correlation between patient age and ΔlogMAR. **(C)** Correlation between patient age and MH diameter.

Subsequent correlation analyses revealed no statistically significant association between the magnitude of visual improvement (ΔlogMAR) and either the preoperative MH diameter (Spearman’s ρ = 0.173, *p* = 0.454) or patient age (ρ = -0.062, *p* = 0.789), as illustrated in Figure 3.

### Safety and complications

No intraoperative or postoperative sight-threatening complications—such as retinal detachment, endophthalmitis, persistent elevated intraocular pressure, or significant intraocular hemorrhage—were observed in any of the 21 eyes during the follow-up period.

### Surgical outcomes summary

A comprehensive summary of surgical outcomes, including visual acuity conversion and anatomical status, is provided in Table 3.

**TABLE 3:** Statistical summary of surgical outcomes.

Variables	Preoperative	Postoperative	statistical test	Effect size (r)
**BCVA**				
logMAR median (IQR)	1.70 (IQR: 1.30–1.70)	0.82 (IQR: 0.61–0.82)	*Z* = −4.022, *p* < 0.001	0.88
Snellen median (range)	20/1,000 (20/2,000–20/200)	20/133 (20/400–20/33)	NA	NA
MH diameter (μm)	717.29 ± 127.00	Closed (100%)	NA	NA

BCVA, best corrected visual acuity; logMAR, logarithm of the minimum angle of resolution; MH, macular hole; IQR, interquartile range.

## Discussion

To improve the closure rate of large MHs, Michalewska et al. introduced the inverted ILM flap technique (15). This approach involves flipping the ILM to cover the MH, thereby promoting the proliferation of glial cells to fill the MH defect, which significantly enhances both anatomical closure rates and postoperative BCVA in idiopathic chronic MHs (9). Nevertheless, conventional inverted or extended ILM peeling techniques often require circumferential removal of the ILM around the macular hole, including the nasal and temporal regions, which may expose the underlying retinal nerve fiber layer and Müller cell endfeet to direct mechanical trauma.

Following the introduction of the inverted ILM flap technique, a growing body of literature has explored alternative ILM flap configurations for large macular holes, aiming to further optimize anatomical closure and flap stability. Notably, Macchi et al. (19) performed a direct comparative study evaluating pedicle transposition flaps, inverted flaps, free ILM flaps, and standard ILM peeling for large full-thickness macular holes. Their results demonstrated that flap-based techniques generally achieved higher anatomical closure rates than standard peeling, while also highlighting important differences in surgical complexity and postoperative flap stability among various flap designs. In particular, free ILM flaps and pedicle transposition flaps were found to be more susceptible to intraoperative manipulation challenges or postoperative displacement, especially in larger defects, owing to the absence of intrinsic structural anchoring. Furthermore, the free ILM flap technique often necessitates the use of perfluorocarbon liquid to stabilize the flap during surgery, which not only increases procedural complexity but also raises surgical costs and potential risks associated with its use.

This comparative evidence underscores that, beyond flap creation itself, the configuration and stability of the ILM scaffold play a critical role in determining surgical success in large and extremely large macular holes.

To address these limitations while preserving the biological rationale of the inverted flap concept, we developed a dual-layer ILM flap technique with superior–inferior (S–I) coverage. Compared with the original inverted ILM flap technique described by Michalewska et al., this approach represents a structured and stability-oriented modification, designed to provide enhanced mechanical support, improved biological scaffolding, and greater resistance to both intraoperative and postoperative flap displacement, particularly in extremely large macular holes.

Specifically, this dual-layer ILM flap technique offers several key advantages over conventional single-layer approaches: (1) Enhanced structural support: The crisscross flap design (superior flap overlapping inferior flap) provides bidirectional scaffolding, reducing tangential traction and minimizing postoperative flap displacement—a critical limitation of single-layer techniques (8). This is particularly evident in our largest MH case (949 μm, Case 20), where complete closure was achieved despite the extreme diameter. (2) Optimized biological microenvironment: The dual-layer configuration may also preserve ILM basement membrane components (e.g., laminin, collagen IV), which are essential for glial cell migration and proliferation (15, 20). (3) Preservation of peri-macular integrity: Unlike extensive ILM peeling techniques, our approach deliberately avoids peeling the ILM on the nasal and temporal sides of the macular hole, thereby maintaining the integrity of the peri-foveal ILM. By limiting ILM removal to the superior and inferior regions used for flap creation, this technique reduces unnecessary manipulation of the inner retinal surface, potentially minimizing trauma to Müller cell endfeet and preserving inner retinal microstructure. This tissue-sparing concept is consistent with emerging strategies that emphasize selective ILM preservation, such as the RONA technique proposed by Nourinia et al. (21), which aims to reduce inner retinal trauma while maintaining a favorable microenvironment for macular hole closure. However, unlike RONA, our technique simultaneously provides direct flap coverage and enhanced mechanical support, which may be particularly advantageous in extremely large macular holes.

In addition to structural reinforcement, optimizing the biological microenvironment at the MH site is crucial for successful healing. Autologous whole blood has emerged as a valuable adjunct in MH surgery due to its dual-action properties (10, 22–24). Mechanically, upon clotting, it acts as a biocompatible and biodegradable adhesive (“bio-glue”), securing tissue flaps (such as the ILM) in place, reducing postoperative displacement, and providing a provisional matrix over the defect (25, 26). Physiologically, it delivers a concentrated source of platelets, growth factors (e.g., platelet-derived growth factor, transforming growth factor-β), fibrinogen, and other cellular components that are pivotal in promoting glial cell chemotaxis, proliferation, and extracellular matrix deposition, thereby facilitating tissue repair and hole closure (9, 27, 28). The rationale for combining autologous blood with the ILM flap technique is synergistic. While the inverted ILM flap provides a structured scaffold, the application of autologous blood enhances flap adherence to the retinal pigment epithelium and surrounding retina, stabilizes the surgical construct during the critical early postoperative phase, and directly delivers biological mediators that accelerate the reparative process. This combination aims to address both the mechanical and biological deficiencies that hinder closure in large and refractory MHs. When combined with the dual-layer ILM flap, autologous whole blood further stabilizes the flaps without necessitating additional ILM peeling, reinforcing both mechanical fixation and biological repair while preserving peri-macular tissue.

Notably, all procedures were performed by a single experienced surgeon, ensuring technical consistency. The 15% C3F8 tamponade combined with strict 2-week prone positioning likely contributed to the high success rate by maintaining stable MH coverage during early healing.

This study demonstrates that the novel dual-layer ILM flap technique with superior-inferior (S-I) coverage combined with autologous blood application achieves 100% anatomical closure in large MHs > 400 μm (mean diameter: 717.3 ± 127.0 μm, range: 521–949 μm), with significant functional improvement (median logMAR BCVA: 1.70 (IQR, 1.30–1.70) → 0.82 (IQR, 0.61–0.82), *p* < 0.001). These results surpass those reported for conventional single-layer ILM flaps and suggest a potential paradigm shift in the surgical management of refractory large MHs.

However, this study has several limitations. First, the retrospective design inherently limits the strength of causal inferences and is subject to potential selection bias, which should be acknowledged as a methodological constraint. Second, the sample size was relatively small (*n* = 21) and derived from a single center, which may restrict generalizability. Third, the absence of a control group treated with alternative surgical techniques prevents direct comparisons of efficacy and safety. Additionally, the minimum follow-up duration of 3 months is relatively short, limiting the assessment of long-term anatomical stability, functional recovery, and delayed complications.

Given these limitations—particularly the retrospective nature of the study as highlighted by the reviewer—our findings should be interpreted as preliminary evidence rather than definitive proof of superiority. Nonetheless, the consistently high closure rate and meaningful visual gains observed in this cohort indicate that the dual-layer ILM flap with S–I coverage combined with autologous whole blood application may be a valuable option for large and otherwise challenging MHs. Future prospective, multicenter randomized controlled trials with extended follow-up are warranted to validate our findings and provide more robust evidence regarding the comparative benefits of this technique.

## Conclusion

The novel dual-layer ILM flap with S-I coverage and autologous blood tamponade is a safe and effective treatment for large MHs (>400 μm), demonstrating 100% anatomical closure and significant visual improvement. While preliminary, these results suggest a paradigm shift for refractory cases. Future randomized trials should compare this technique to single-layer flaps and APC adjuncts.

## Data Availability

The original contributions presented in the study are included in the article/supplementary material, further inquiries can be directed to the corresponding authors.

## References

[1] Ho AC Guyer DR Fine SL. Macular hole. *Surv Ophthalmol.* (1998) 42:393–416. 10.1016/s0039-6257(97)00132-x 9548570

[2] Duker JS Kaiser PK Binder S de Smet MD Gaudric A Reichel E The International Vitreomacular Traction Study Group classification of vitreomacular adhesion, traction, and macular hole. *Ophthalmology.* (2013) 120:2611–9. 10.1016/j.ophtha.2013.07.042 24053995

[3] Kelly NE Wendel RT. Vitreous surgery for idiopathic macular holes. Results of a pilot study. *Arch Ophthalmol.* (1991) 109:654–9. 10.1001/archopht.1991.01080050068031 2025167

[4] Gass JD. Reappraisal of biomicroscopic classification of stages of development of a macular hole. *Am J Ophthalmol.* (1995) 119:752–9. 10.1016/s0002-9394(14)72781-3 7785690

[5] Gonder JR Proulx AA Gale JS. Anatomic and visual outcomes after indocyanine green-assisted peeling of the retinal internal limiting membrane in idiopathic macular hole surgery. *Am J Ophthalmol.* (2004) 138:690–1. 10.1016/j.ajo.2004.06.044 15488824

[6] Beutel J Dahmen G Ziegler A Hoerauf H. Internal limiting membrane peeling with indocyanine green or trypan blue in macular hole surgery: a randomized trial. *Arch Ophthalmol.* (2007) 125:326–32. 10.1001/archopht.125.3.326 17353402

[7] Steel DH Donachie PHJ Aylward GW Laidlaw AH Williamson TH Yorston D Factors affecting anatomical and visual outcome after macular hole surgery: findings from a large prospective UK cohort. *Eye (Lond).* (2021) 35:316–25. 10.1038/s41433-020-0844-x 32231259 PMC7852599

[8] Gu C Qiu Q. Inverted internal limiting membrane flap technique for large macular holes: a systematic review and single-arm meta-analysis. *Graefes Arch Clin Exp Ophthalmol.* (2018) 256:1041–9. 10.1007/s00417-018-3956-2 29532170

[9] Lai CC Chen YP Wang NK Chuang LH Liu L Chen KJ Vitrectomy with internal limiting membrane repositioning and autologous blood for macular hole retinal detachment in highly myopic eyes. *Ophthalmology.* (2015) 122:1889–98. 10.1016/j.ophtha.2015.05.040 26143541

[10] Xu Z Wang Y Chen Y. Effect of autologous whole blood in surgery for full-thickness macular hole: a propensity score matching analysis. *BMC Ophthalmol.* (2025) 25:173. 10.1186/s12886-025-04019-6 40197248 PMC11974085

[11] Wakely L Rahman R Stephenson J. A comparison of several methods of macular hole measurement using optical coherence tomography, and their value in predicting anatomical and visual outcomes. *Br J Ophthalmol.* (2012) 96:1003–7. 10.1136/bjophthalmol-2011-301287 22611137

[12] Ullrich S Haritoglou C Gass C Schaumberger M Ulbig MW Kampik A. Macular hole size as a prognostic factor in macular hole surgery. *Br J Ophthalmol.* (2002) 86:390–3. 10.1136/bjo.86.4.390 11914205 PMC1771090

[13] Baumann C Almarzooqi A Blobner K Zapp D Kirchmair K Schwer LS Repeatability and reproducibility of macular hole size measurements using optical coherence tomography. *J Clin Med.* (2021) 10:2899. 10.3390/jcm10132899 34209752 PMC8268292

[14] Rossi T Bacherini D Caporossi T Telani S Iannetta D Rizzo S Macular hole closure patterns: an updated classification. *Graefes Arch Clin Exp Ophthalmol.* (2020) 258:2629–38. 10.1007/s00417-020-04920-4 32910308

[15] Michalewska Z Michalewski J Adasiewicz-Barniak K Nawrocki J. Inverted internal limiting membrane flap technique for large macular holes. *Ophthalmology.* (2010) 117:2018–25. 10.1016/j.ophtha.2010.02.011 20541263

[16] Bailey IL Lovie JE. New design principles for visual acuity letter charts. *Am J Optom Physiol Opt.* (1976) 53:740–5. 10.1097/00006324-197611000-00006 998716

[17] Holladay JT. Proper method for calculating average visual acuity. *J Refract Surg.* (1997) 13:388–91. 10.3928/1081-597X-19970701-16 9268940

[18] Kakinoki M Araki T Iwasaki M Ueda T Sano H Hirano Y Surgical outcomes of vitrectomy for macular hole retinal detachment in highly myopic eyes: a multicenter study. *Ophthalmol Retina.* (2019) 3:874–8. 10.1016/j.oret.2019.04.026 31257070

[19] Macchi I Huelin FJ Young-Zvandasara T Di Simplicio S Kadhim MR Chawla H Pedicle transposition flap, inverted flap, free flap, and standard peel for large full-thickness macular holes: a comparative study. *Retina.* (2024) 44:1552–9. 10.1097/IAE.0000000000004142 39073100

[20] Teng Y Zhang X. Temporal inverted internal limiting membrane flap technique for myopic macular hole retinal detachment reconstruction. *J Int Med Res.* (2024) 52:3000605231223635. 10.1177/03000605231223635 38235655 PMC10798096

[21] Nourinia R Nikzad P Abolhosseini M Moshtaghion SM Abtahi SH. RONA technique: a novel ILM peeling method for treatment of large full-thickness macular holes. *Retina.* (2023) 43:692–7. 10.1097/IAE.0000000000003390 34954779

[22] Zhu K Wang Y Lei B Chen L Zhang Y Chang Q Comparison of the inverted internal limiting membrane flap technique without versus with an autologous blood clot for treating macular hole-associated retinal detachment. *Eye Vis (Lond).* (2025) 12:1. 10.1186/s40662-024-00417-x 39743620 PMC11694389

[23] Peng J Chen C Jin H Zhang H Zhao P. Autologous lens capsular flap transplantation combined with autologous blood application in the management of refractory macular hole. *Retina.* (2018) 38:2177–83. 10.1097/IAE.0000000000001830 29045320

[24] Purtskhvanidze K Frühsorger B Bartsch S Hedderich J Roider J Treumer F. Persistent full-thickness idiopathic macular hole: anatomical and functional outcome of revitrectomy with autologous platelet concentrate or autologous whole blood. *Ophthalmologica.* (2018) 239:19–26. 10.1159/000481268 29050013

[25] Wu AL Chuang LH Wang NK Chen KJ Liu L Yeung L Refractory macular hole repaired by autologous retinal graft and blood clot. *BMC Ophthalmol.* (2018) 18:213. 10.1186/s12886-018-0898-8 30157808 PMC6114829

[26] de Azeredo Bastos TM Neves LL Freitas LP Isaac DLC Casella AMB Ávila MP. New surgery technique for refractory macular hole guided by intraoperative OCT: free internal limiting membrane flap and autologous blood clot. *Int J Retina Vitreous.* (2025) 11:60. 10.1186/s40942-025-00681-6 40426226 PMC12107886

[27] Anitua E Andia I Ardanza B Nurden P Nurden AT. Autologous platelets as a source of proteins for healing and tissue regeneration. *Thromb Haemost.* (2004) 91:4–15. 10.1160/TH03-07-0440 14691563

[28] Werner S Grose R. Regulation of wound healing by growth factors and cytokines. *Physiol Rev.* (2003) 83:835–70. 10.1152/physrev.2003.83.3.835 12843410

